# The occurrence and development mechanisms of esophageal stricture: state of the art review

**DOI:** 10.1186/s12967-024-04932-2

**Published:** 2024-01-31

**Authors:** Fang Yang, Yiwei Hu, Zewen Shi, Mujie Liu, Kefeng Hu, Guoliang Ye, Qian Pang, Ruixia Hou, Keqi Tang, Yabin Zhu

**Affiliations:** 1 Health Science Center, Ningbo University, Ningbo, 315211 People’s Republic of China; 2 The First Affiliated Hospital of Ningbo University, Ningbo, 315000 People’s Republic of China; 3 Institute of Mass Spectrometry, School of Material Science and Chemical Engineering, Ningbo University, Ningbo, 315211 People’s Republic of China; 4 Ningbo No.2 Hospital, Ningbo, 315001 People’s Republic of China

**Keywords:** Esophagus, Stricture, Biological mechanism, Occurrence, Development

## Abstract

**Background:**

Esophageal strictures significantly impair patient quality of life and present a therapeutic challenge, particularly due to the high recurrence post-ESD/EMR. Current treatments manage symptoms rather than addressing the disease's etiology. This review concentrates on the mechanisms of esophageal stricture formation and recurrence, seeking to highlight areas for potential therapeutic intervention.

**Methods:**

A literature search was conducted through PUBMED using search terms: esophageal stricture, mucosal resection, submucosal dissection. Relevant articles were identified through manual review with reference lists reviewed for additional articles.

**Results:**

Preclinical studies and data from animal studies suggest that the mechanisms that may lead to esophageal stricture include overdifferentiation of fibroblasts, inflammatory response that is not healed in time, impaired epithelial barrier function, and multimethod factors leading to it. Dysfunction of the epithelial barrier may be the initiating mechanism for esophageal stricture. Achieving perfect in-epithelialization by tissue-engineered fabrication of cell patches has been shown to be effective in the treatment and prevention of esophageal strictures.

**Conclusion:**

The development of esophageal stricture involves three stages: structural damage to the esophageal epithelial barrier (EEB), chronic inflammation, and severe fibrosis, in which dysfunction or damage to the EEB is the initiating mechanism leading to esophageal stricture. Re-epithelialization is essential for the treatment and prevention of esophageal stricture. This information will help clinicians or scientists to develop effective techniques to treat esophageal stricture in the future.

## Introduction

The esophagus is a canal extending from the pharynx to the stomach and transporting food and water from mouth to stomach [1, 2]. Histologically, the esophagus can be divided into four architectural layers in cross-section; mucosal, submucosal, muscular, and extima. The mucosal layer is subdivided into the epithelium, composed of non-keratinized stratified squamous epithelial cells, the lamina propria, and the mucosal muscle. The esophagus muscle contains the inner circular and outer longitudinal muscular bilayers, consisting of skeletal muscle cells at the upper one-third and smooth muscle cells at the bottom one-third length with the mixture in the middle. The stability of the internal esophageal environment is crucial for the normal esophagus[3, 4].

**Fig. 1:**
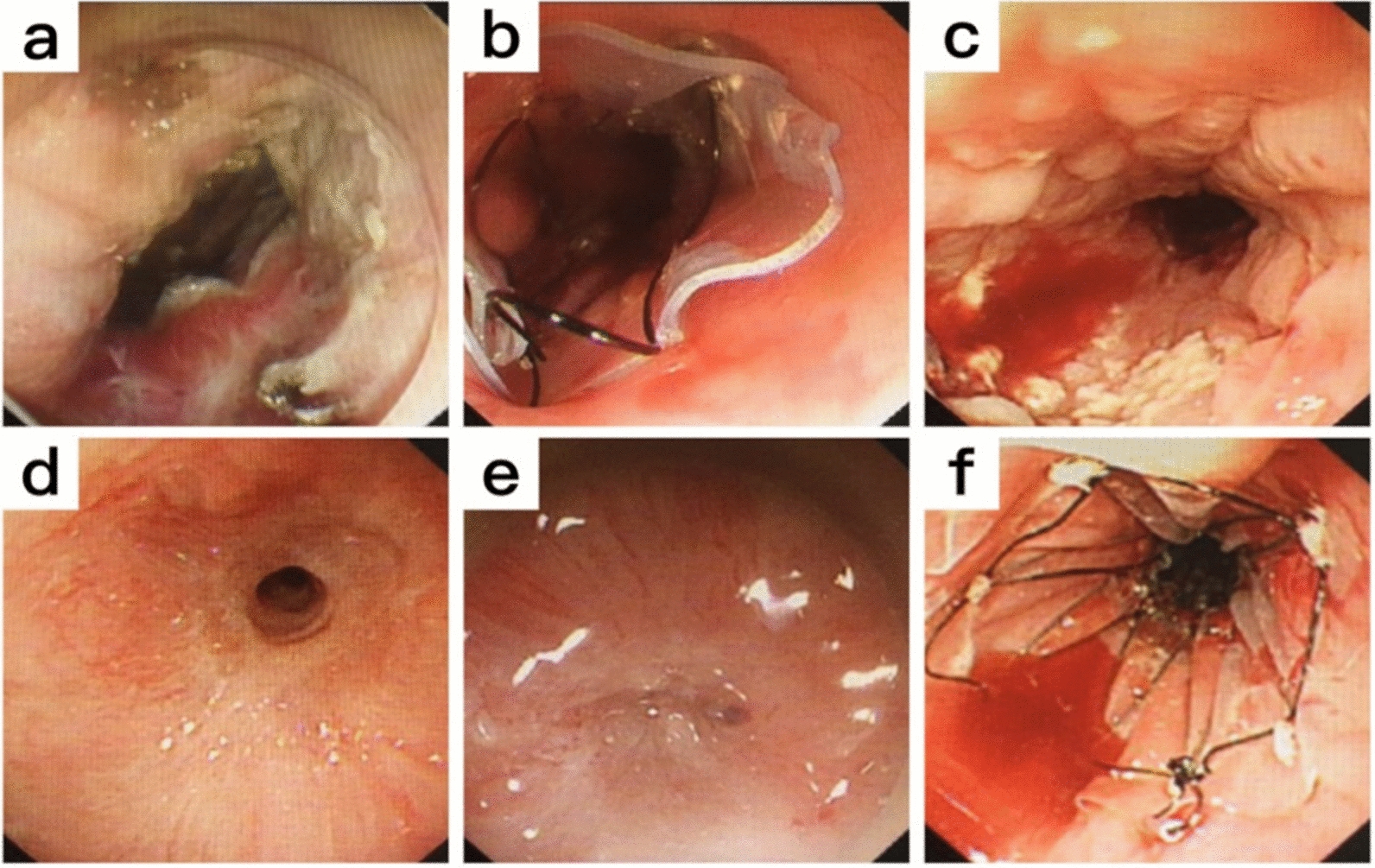
The stricture case after endoscopic submucosal dissection (ESD) surgery due to early-stage esophageal cancer. **a** Postoperative wound; **b** metal stents were placed towards preventing stricture; **c** the stent was removed after two months post-operation; **d** stricture recurred 20 days after stent removal; **e** completely blocked one and a half months after stent removal; **f** surgical stent placement again. The case was provided by the Department of Gastroenterology of Hospital Affiliated with Ningbo University School of Medicine

With the development of contemporary endoscopic techniques, from ordinary endoscopic mucosal resection (EMR) to endoscopic submucosal dissection (ESD) [5], more and more esophageal diseases can be treated with endoscopic technology [6–8]. Figure 1 shows a typical clinical esophageal stricture case (provided by the Department of Gastroenterology of the Hospital Affiliated to School of Medicine, Ningbo University). The progression of postoperative esophageal strictures is torture for both patients and clinicians. The literature reports that resection of more than 3/4 circumference of the esophageal mucosa with ESD is a high-risk factor for esophageal stricture [9], with an incidence of 100% and 56–76% for esophagotomy and non-esophagotomy, respectively [10–15].

In recent years, with the development of genomics and proteomics, the biological mechanisms leading to esophageal strictures are becoming more apparent. However, there are still many mysteries that remain to be solved. Some scholars believe that excessive tissue fibrosis is closely related to esophageal strictures and that the biological mechanisms leading to esophageal fibrosis may be an entry point for esophageal strictures. Topical use of Mitomycin C (MMC), a chemotherapeutic agent that inhibits fibrosis, at esophageal strictures can reduce the number of physical dilatations and give evidence of excessive fibrotic mechanisms leading to esophageal strictures [16].

Some scholars believe that esophageal stricture is associated with the excessive inflammatory response in the injured local tissues. A timely inflammatory response due to immune stress is beneficial for organism repair. Still, an excessive inflammatory response will be accompanied by a fractional secretion of inflammatory factors by immune cells and somatic cells, eventually leading to esophageal stricture. Steroids, clinically representative as anti-inflammatory therapy, are currently the main therapeutic agents for the prevention and treatment of esophageal strictures because oral administration with steroids before surgery and/or local injection in the postoperative region have some inhibitory effect on esophageal strictures [15, 17–19]. The usage of steroids gives supporting evidence that the inhibition of inflammatory response might be the mechanism of the occurrence of esophageal stricture. However, the efficiency of oral feeding or local injection is not the same for all esophageal patients; there are significant differences in patients with diverse disease seriousness or sensitivity to steroids.

Currently, some scholars focus on the loss of esophageal epithelial barrier function that ultimately leads to esophageal stricture [20–23]. The proposed mechanism gives a new direction to the mechanism of esophageal stricture. Under the influence of some microenvironments, the expression of genes and proteins of esophageal epithelial cells is altered, leading to the dysfunction of the esophageal epithelial barrier and eventually esophageal stricture [24–31]. With the progress of research in tissue engineering, the re-epithelialization of the esophagus is sought to be accomplished by biomaterial-assisted cells (e.g., cell patches, etc.) [32–34], thus treating esophageal strictures. The therapeutic approach of tissue engineering argues for the stricture's mechanism [35–41].

Until now, we have found that neither the hyper-fibrosis, the inflammatory response, nor even barrier damage can explain the mechanisms of esophageal strictures at a single level. In the clinic, neither treatments of repeat endoscopic dilation, steroid administration, etc. nor tissue engineering technologies like biological scaffolds can fundamentally solve the problem of esophageal stricture. The recurrence and repeated dilation are unavoidable. In recent years, the mechanism of esophageal stricture has been detected with the advances in molecular biology, genomics, and proteomics, though it is still unclear. Therefore, it is necessary to clarify the biological mechanisms of esophageal stricture so as to develop clinic therapies for this disease. This review aims to detect the possible molecular mechanisms and the key factors leading to esophageal stricture, further providing probable molecular targets or novel methods for treating the stricture.

## Biological mechanisms of esophageal stricture

### Inflammatory response

The inflammatory response is a typical defense of the human body against external stimuli such as bacteria, viruses, or other antigens. A timely and moderate inflammation can efficiently remove harmful antigens and allow the body to recover. However, incomplete removal of harmful antigens or the persistent inflammatory response will transform acute inflammation into chronic inflammation, inducing the secretion of many cytokines and profibrotic factors. In the case of esophageal disease, chronic inflammation will cause progressive or even excessive fibrosis, excessive deposition of extracellular matrix (ECM) [42–44], and eventually lead to the occurrence of stricture. The inflammatory response requires a high degree of cooperation among all systems in the body, of which the immune system takes the lead and plays a vital role inefficiently clearing harmful antigens.

**Fig. 2:**
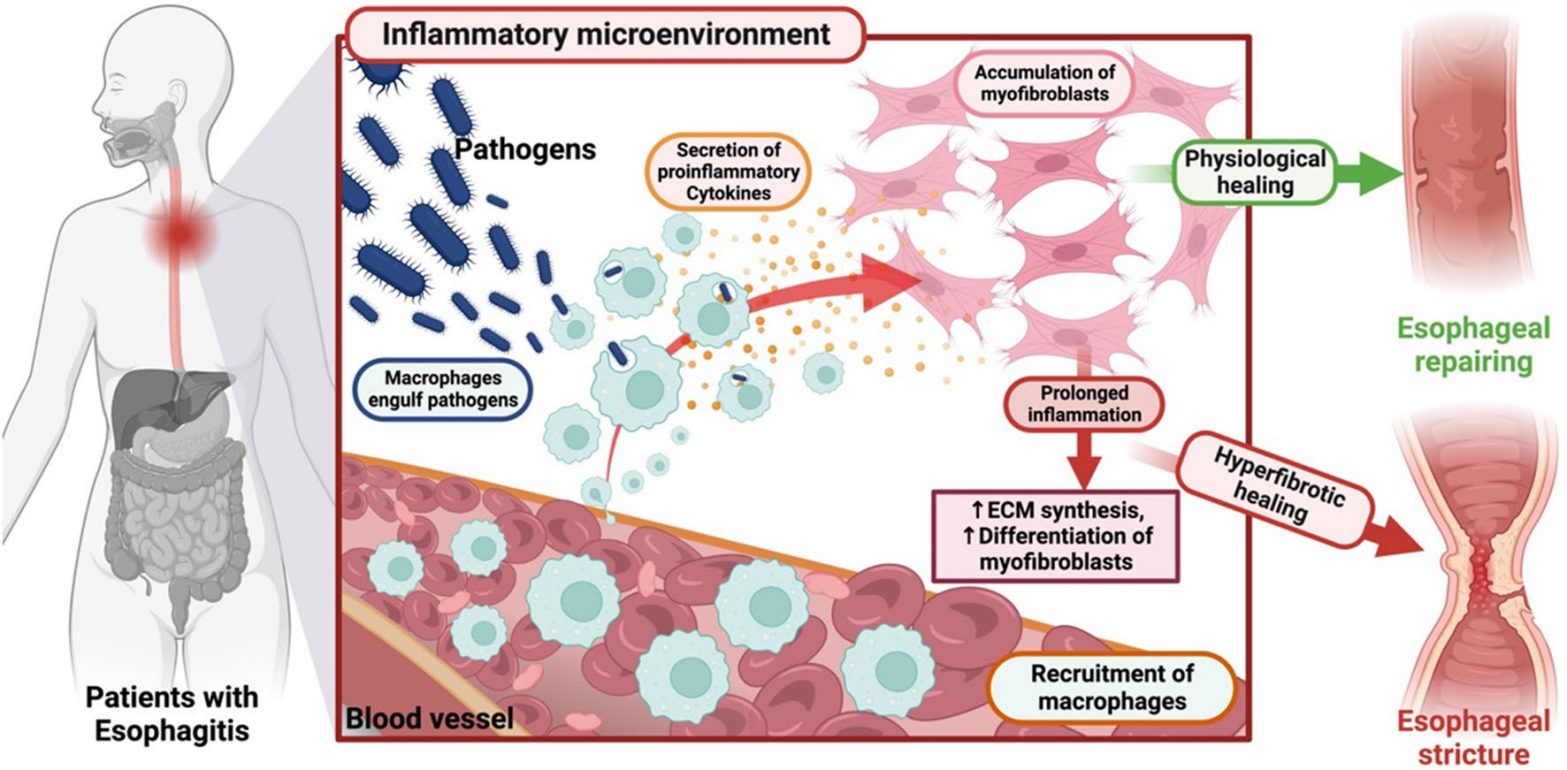
Relationship between macrophages and fibroblasts in the inflammatory response during esophageal physiological healing and hyperfibrotic healing

The activation of the immune system often begins with phagocytosis of macrophages [45], stronger phagocytosis shorter duration of the disease [46]. The critical role of macrophages is to balance the inflammatory regression and fibrotic progress [47, 48]. Local stimulation or invasion of harmful antigens induces the migration of macrophages, which gradually move toward the center of inflammation occurrence. At the same time, myofibroblasts emerge, both generating complex-forming signaling and close intercellular communication [49–51]. Macrophages secrete a variety of cytokines, including pro-fibrotic cytokines like transforming growth factor (TGF-β), which in turn promote the occurrence of fibrosis [52, 53]. Effective inflammatory response, accompanied by rapidly recruiting macrophages that phagocytose pathogens and secrete cytokines to act on fibroblasts, promotes inflammatory healing and wound repair. In the esophagus as well, when inflammation is not healed in a timely manner, pro-inflammatory factors lead to over-differentiation of fibroblasts into myofibroblasts, which in turn results in an accumulation of ECM, leading to tissue fibrosis and ultimately esophageal stricture (Fig. 2).

Macrophage acts as a “bridge” from inflammation to fibrosis-related diseases. They are involved in all phases of the fibrotic progress, with various roles and phenotypes as the fibrosis develops [54–56]. The phagocytosis of macrophages has an inhibitory effect on the fibrotic phenotype [57]. Several studies verified that macrophages can shift themselves into myofibroblasts directly, exacerbating the occurrence of fibrosis and accelerating fibrosis-related disease in some pathological cases, for example, renal fibrosis [58–63].

In esophagus-related diseases, the inflammatory bio-condition of the organ seems to be gradual and severe progress, from the beginning of gastroesophageal reflux where gastric acid or H^+^ions coming from foods constantly stimulate the mucosa tissue of the esophagus to produce damage and inflammatory stress response, to the pathological changes in the epithelium to cause diseases like Barrett's esophagus (BE) and even tumor occurrence. It can be found that the inflammatory response is in the progression of every stage of esophageal diseases. With inflammatory infiltration, the expression of critical genes about the activity and development of normal cells, such as *BMP4* and *PTGS2*, etc., alters abnormally [64–67].

Esophagitis is the most common disease of esophagus in clinics. Eosinophilic esophagitis (EoE) is the relatively specific one among inflammatory-related diseases. The progression often varies with age, manifesting as an inflammatory response in children and as fibrosis or/and esophageal stricture in adults [68, 69]. Allergens, excess cytokines, or antigens leading to Eosinophilia in local tissues are the main pathways of EoE [70–73]. For example, Eosinophilia was developed in mice infected by Aspergillus fumigatus, which was verified that eosinophil accumulation and collagen deposition were mainly associated with interleukin-5 (IL-5) [74]. This allergic reaction produces various cytokines and mediators, causing diffuse esophagitis, esophageal hyperhidrosis, and eventually esophageal stricture. This may explain that EoE presents as inflammation in children but hyperfibrosis and esophageal stricture in adults with the disease progression.

The present treatment in the clinic, oral medication or steroid injection, should support this inflammatory mechanism. Steroids are standard medicine adopted to moderate inflammation. Injection of steroid medicine like triamcinolone acetate into the focal infected area to be operated on [75]. This prophylactic steroid injection has been applied as one method to prevent esophageal stricture after the ESD surgery [13, 19]. Ramage et al*.* reported that steroid injection combined with balloon dilatation significantly reduced the dilatation frequency from 60 to 13% (*p* < 0.01) for recurrent dysphagia patients with esophageal stricture. The recurrence of esophageal stricture was delayed from 9 to 15 months(*p* < 0.01) [76]. In a randomized controlled trial (RCT), Takahashi et al. explored the therapeutic effects of steroid use for esophageal strictures. Thirty-two patients with mucosal defects involving ≥ 75% of the esophageal circumference were randomized to treatment with local injection of steroids (n = 16) and conventional treatment (n = 16). The results revealed a significant reduction in the number of re-dilatation procedures in the group treated with steroids, but a five-significant difference in the frequency of upper esophageal strictures. This reveals that prophylactic endoscopic steroid injection appears to be a safe means of relieving the severity of esophageal stricture following extensive ESD [14]. However, it is debatable till now whether it is necessary to administer a specific steroid dose, how often injections should be performed, and what’s the efficiency to prevent the stricture.

Undoubtedly, the inflammatory responses are incremental in the progression of esophageal disease. Once acute inflammation is not cured in time, it will turn into chronic inflammation. Chronic inflammation causes the mucosa tissue to fluctuate frequently between damage and repair, leading to hyperfibrosis occurrence; finally, esophageal stricture appears. The limited treatments against esophageal stricture, for example, local injection of steroids, take only partial effects, incompletely inhibiting inflammation and fibrosis. Thus, controlling the inflammatory response alone in response to hyperfibrosis is often insufficient and portends that intervening in the process of fibrosis progression is also particularly important, as discussed in the next section.

### Excessive differentiation of fibroblasts

**Fig. 3:**
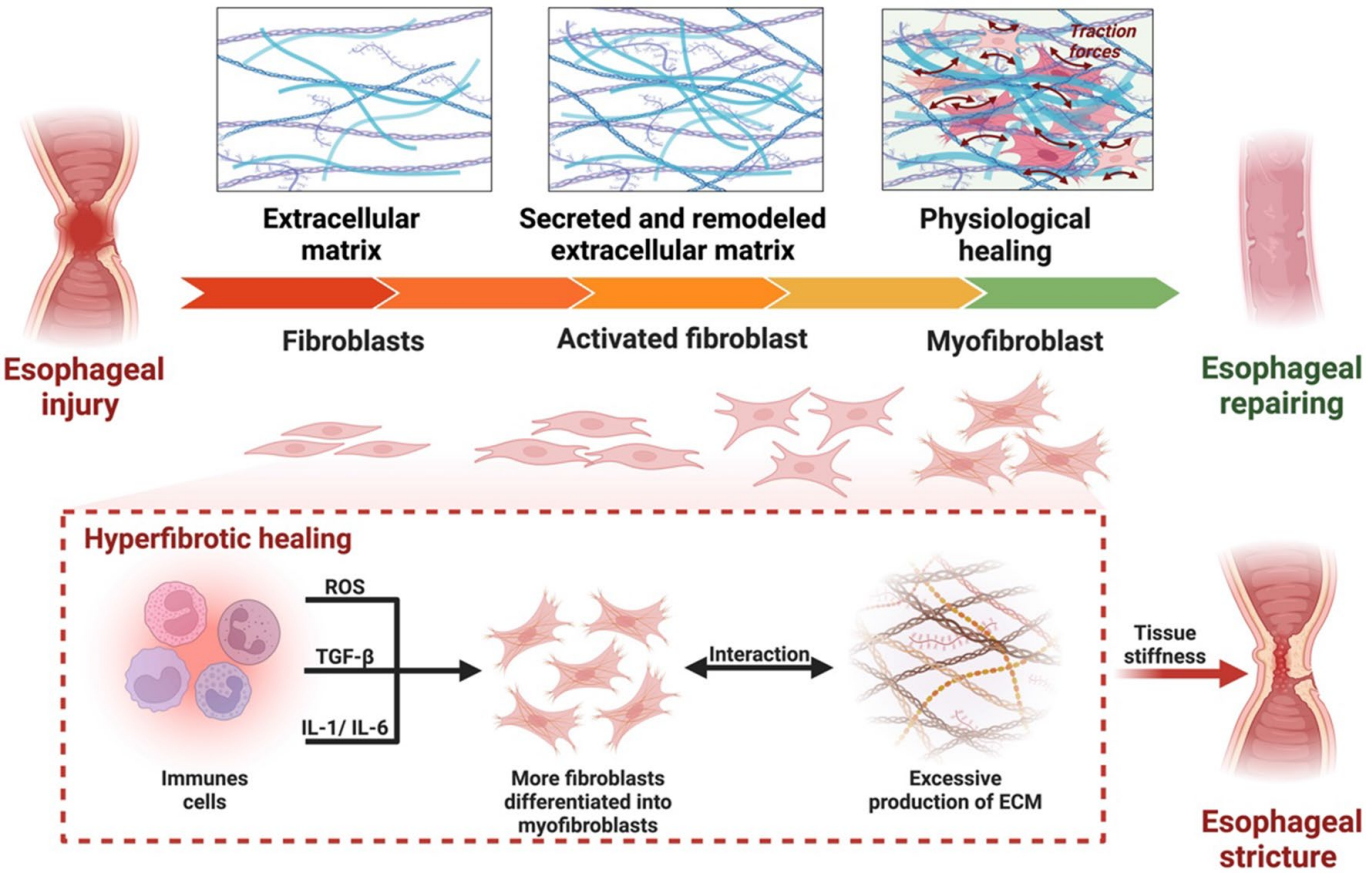
Transformation of fibroblasts and extracellular matrix during esophageal physiological healing and hyperfibrotic healing

Fibroblast exists in most tissues and organs of the human body with a normal spindle shape. It plays an essential role in the secretion of ECM and the formation of granulation. When the tissue is injured, it takes part in wound healing through secreting ECM to give the body good "soil" and creating new connective tissue by depositing fibers, elastin, and laminin. In this case, the fibroblast undergoes a differentiation to become myofibroblast to promote organism repair when the homeostatic environment is disrupted, or the organism is under stressful situations [77–79]. The balance of matrix metalloproteinases (MMPs) and the inhibitors of MMPs in the microenvironment can remodel the structure of the ECM that is excessively secreted by myofibroblasts. Regular repair is accompanied by fibroblast differentiation and significant but not excessive ECM secretion, which play a critical role in the body during physiological healing (Fig. 3). Due to the altered microenvironment at the injury site, the emergence of various immune cells and the secretion of cytokines and ECM will promote fibroblasts to differentiate into myofibroblasts. Pakshir*et al*. found that myofibroblasts promote tissue repair by excessively expressing α-smooth muscle actin (α-SMA) to recruit myofibroblasts onto large amounts of ECM [80]. In research, the targeted transformation from fibroblasts to myofibroblasts is often determined by fluorescently labeling α- SMA [81].

Although myofibroblasts are particularly important in physiological repair, their roles are different at different periods in physiological healing. In chronic wound or chronic inflammation, the shift from fibroblast to myofibroblast can lead passively to excessive deposition of ECM and disorganized accumulation of cells, ultimately leading to hyperfibrotic healing, which manifests as esophageal stricture in the esophagus (Fig. 3) [16, 81–83]. According to epidemiological statistics, organ dysfunction due to fibrotic disease eventually leads to the death of about 45% of patients in the developed countries [84–86]. Hyperfibrosis in fibrotic disorders arises through the interplay of numerous biomarkers and molecular targets. These biomarkers (chemokines, pro-inflammatory factors, TGF-β superfamily, etc.) ultimately lead to organ or tissue hyperfibrosis by participating in different signaling pathways (Table 1). Specifically, the chemokine CCL2/MCP-1 is implicated in provoking an inflammatory response that leads to the over-differentiation of fibroblasts, a key event in the pathogenesis of fibrosis. Moreover, these biomarkers, which are instrumental in the progression of fibrosis, may also be exploited therapeutically to impede this process. For instance, Cenicriviroc, a selective inhibitor of CCL2/MCP-1, demonstrates anti-fibrotic properties by inhibiting the recruitment of macrophages into the local inflammatory environment [87, 88].

**Table 1:** Selected biomarkers of mechanisms leading to excessive fibrosis

Biomarkers/Targets	Classification	Substance/Intervention	Findings/Mechanism
CCL2/MCP-1	Chemokines	Cenicriviroc [87, 88]	CCL2/MCP-1 leads to excessive fibrosis by promoting inflammatory responses, inducing fibroblast proliferation and differentiation, and mediating extracellular matrix remodeling [100]
TNF-$\alpha$, IL-1, IL-6	Pro-inflammatory cytokines	Glucocorticoid	These pro-inflammatory cytokines can act synergistically to promote fibrosis by stimulating fibroblast activation, collagen production, and inflammation [101–103]
TGF-$\beta$	TGF-β superfamily	Pirfenidone [104, 105]	TGF-β leads to excessive fibrosis by activating fibroblasts, promoting extracellular matrix synthesis, regulating inflammatory response, and inhibiting apoptosis [92, 106]
VEGF-R, PDGF-R, FGF-R	Receptor Tyrosine Kinases	Nintedanib [107, 108]	Excessive activation of these receptors can lead to proliferation and differentiation of fibroblasts, as well as excessive synthesis and deposition of extracellular matrix proteins, leading to the development of fibrosis. In addition, the activation of these receptors can also cause the infiltration and activation of inflammatory cells, further aggravating the fibrotic process
FXR	Steroid/steroid hormone receptor superfamily	Obeticholic acid	FXR activation exerts anti-fibrotic effects by reducing inflammation, modulating TGF-β signaling [109, 110]
ROS	/	Machine perfusion [111]	ROS is involved in the occurrence and development of excessive fibrosis through mechanisms such as oxidative stress, inflammation, apoptosis and necrosis, oxidative protein modification, and fibrocyte activation [112]

*CCL2/MCP-1* chemokine (C–C motif) ligand 2/monocyte chemoattractant protein-1, *TNF-*$\alpha$ tumor necrosis factor-$\alpha$, *IL* interleukin, *TGF-*$\beta$ transforming growth factor-$\beta$, *VEGF-R* vascular endothelial growth factor receptor, *PDFG-R* platelet-derived growth factor receptor, *FGF-R* fibroblast growth factor receptor, *FXR* Farnesoid X Receptor, *ROS* reactive oxygen species

Histologically, the main layers of esophageal stricture caused by the esophageal disease are in the muscularis mucosa and submucosa, where the conversion of fibroblasts to myofibroblasts is undoubtedly essential in case of damage. It can contribute to the repair of damaged tissue. However, as mentioned above, persistent pathological activation of myofibroblast transformation will lead to the development of esophageal fibrosis and even tumorigenesis [70]. Fibroblast-to-myofibroblast shift is mainly mediated by activation of TGF-β pathway [89, 90], followed by activation of TGF-β receptor downstream molecules like Smads, JunD [91, 92], and other interconnected signaling pathways such as platelet-derived growth factor pathway (PDGF) and receptor tyrosine kinase pathways (RPTKs) [93–96]. These downstream receptors and signaling molecules eventually lead to fiber-derived cell proliferation, motility, secretion of ECM and cellular morphological transformation, and even cell mis-differentiation [97–99]. The esophageal fibrogenic cells (predominantly myofibroblasts) in the mucosal muscle and submucosa eventually lead to excessive fibrosis, stiffness, and finally stricture of the esophagus.

In the clinic, local injections of MMC are employed to treat recurrent esophageal strictures. MMC is a chemotherapeutic reagent applied topically to inhibit fibrosis towards curing post-surgical scarring [113–116]. In a rigorously designed double-blind trial, El-Asmar et al. explored the efficacy of MMC for esophageal stricture management. Forty patients from their medical center, spanning from January 2008 to October 2010, were randomly split into two groups: one receiving MMC and the other a placebo. Post six months of monitoring and assessment, the MMC group demonstrated an 80% success rate in stricture resolution, significantly surpassing the placebo group's 35% improvement. Additionally, the MMC group averaged fewer dilation procedures (n = 3.85 ± 2.08) compared to the placebo recipients (n = 6.9 ± 2.12), highlighting MMC's role in reducing the need for repetitive treatments for those suffering from esophageal strictures [16]. Findings from this clinical trial reveal that the local application of MMC, which inhibits the excessive differentiation of fibroblasts, can partly impede the progression of esophageal stricture. This indicates that the over-differentiation of fibroblasts contributes to esophageal stricture. Because the esophagus serves as an elongated passage to the external world, it is particularly critical to avert the excessive differentiation of fibroblasts by exogenous factors, a subject further explored in the next section on the EEB [20].

### Damage of the esophageal epithelial barrier (EEB)

**Fig. 4:**
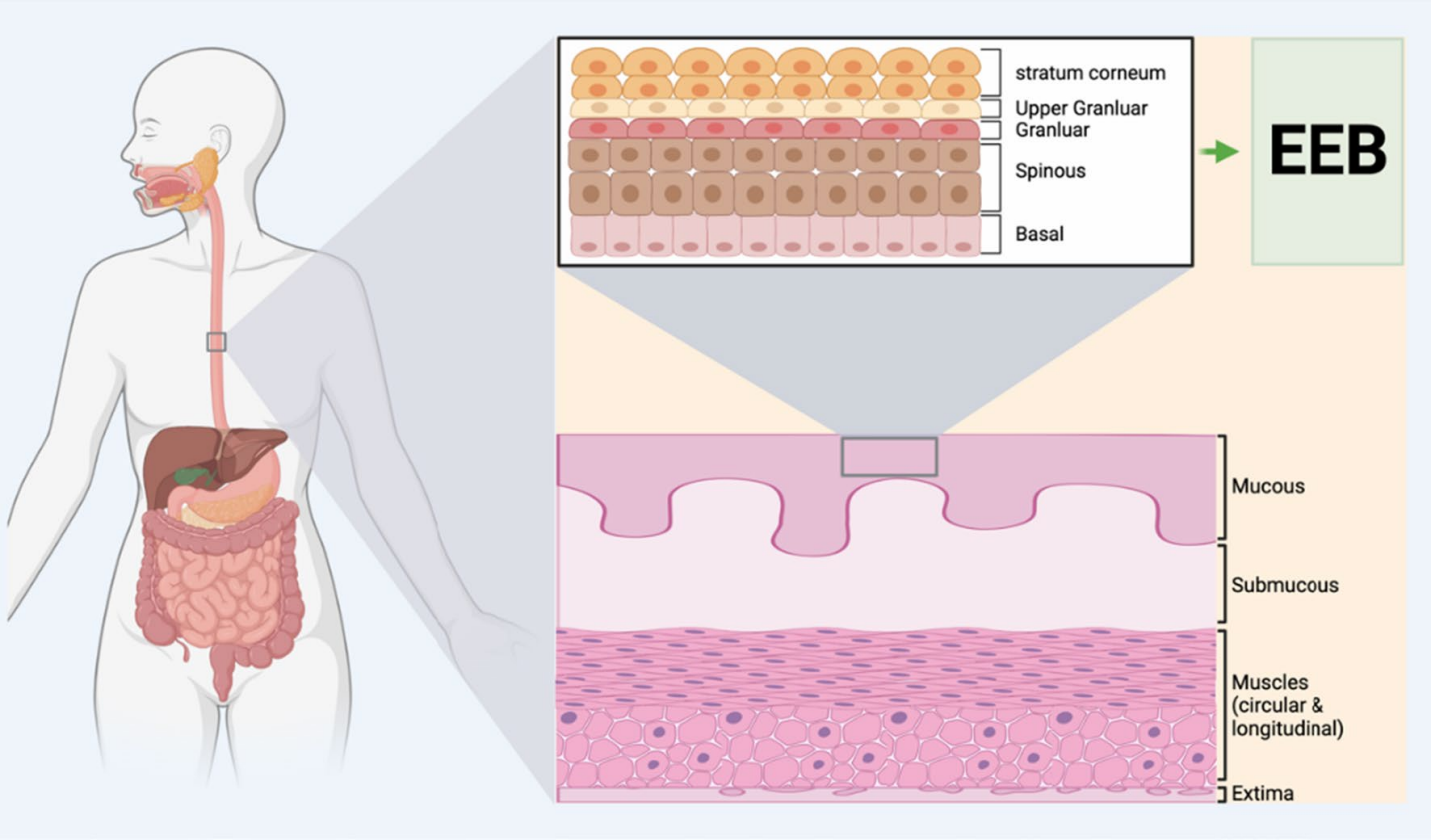
Schematic diagram of the Esophageal epithelial barrier (EEB) structure in the esophagus

The epithelium of the esophagus consists of stratified nonkeratinized squamous epithelial cells. The cells synthesize keratinized envelope proteins to form the intact EEB structure, as shown in Fig. 4. This EEB can effectively block the attack of foreign antigens and allergens, H^+^ ions, etc., to protect the inner tissue [117–119]. Many kinds of esophageal diseases result from the damage of this EEB structure [120, 121]. However, few studies show how EEB damage leads to diseases. Simultaneously, the biological mechanisms of esophageal stricture related to EEB damage have been poorly investigated.

Keratin 14 (*KRT 14*) and filaggrin (*FLG*) are important proteins in the formation of the cornified envelope in the esophageal epithelial cells of the EEB structure. E-cadherin (*E-cad*) and Zonula Occludens (*ZO-1*) are important proteins for the esophageal epithelial cell–cell junctions in the EEB structure. They work together to maintain the normal function of the EEB [122–127]. A significant decrease of these proteins would result in spongy-like loosening among epithelial cells, increasing the intercellular gap and weakening the barrier function of EEB [128]. The enlarged gaps between epithelial cells in the patients with reflux esophagitis were once observed by laser confocal microscopy, which verified the theory about the relationship between EEB function and esophagitis [129]. Because the enlargement of the epithelial cell gaps will let H^+^ ions and other antigenic substances from diets or gastric reflux cross the EEB and invade the mucosa or/and submucosa, an allergic reaction or inflammatory response is triggered, ultimately leading to the occurrence of esophagitis [128] or BE [130–132]. These findings provide clues to the link between the damage of EEB structure and the occurrence of stricture diseases.

EEB dysfunction occurs in many pathologic conditions, including EoE, gastroesophageal reflux disease (GERD), and BE. The normal esophagus maintains proper spaces and junctions among epithelial cells. Proteins like ZO-3, ZO-1, and filaggrin are highly expressed [128]. However, in the diseased esophagus, dilatation of intercellular spaces is a prominent feature observed on light or under electron microscopy. The expressions of those proteins are reduced significantly. Using the transmission electron microscope (TEM), investigators have found the dilated intercellular space and thus documented it as a sensitive marker in patients with GERD and BE [131]. Once the GERD or the BE recurs, the eventual outcome is the esophageal stricture.

The mucosal ulcers cause EEB damage. Many specific cytokines, such as insulin growth factor-1 (IGF-1) and platelet-derived growth factor-C (PDGF-C) etc. will be produced [132]. These cytokines induce myofibroblasts to secrete large amounts of collagen to fill the tissue defect at the site of the ulcer and consequently lead to scar formation. Though the scar or/and ulcer have been considered positively correlated with esophageal stricture, there are limited studies to demonstrate that the damage of EEB is associated with the development of esophageal stricture disease. Still, the exact relationship between EEB structure, esophageal stricture formation, and the critical molecular mechanisms remain unclear.

We consider that the stability of EEB structure plays a vital barrier role in the esophagus. Genes like transglutaminase 1(*TGM1*), cystatin A(*CSTA*), transglutaminase 3(*TGM3*), involucrin(*IVL*), and loricrin (*LOR*) mediate the regular expression of proteins for the cornified envelope, thereby maintaining the EEB struction and function [117]. The abnormalities in genes will affect the normal synthesis of the cornified envelope proteins, further weakening the cell–cell junction. This pathology was discovered in the diseases like skin tissue [133–138]. However, these genes regulating barrier function have not been reported in the esophagus.

The clinical administrations, including steroid injections, stents, or dilators implantation, have had unsatisfactory efficiency for esophageal stricture. Tissue engineering techniques appear to be necessary for mucosal regeneration and EEB repairing. More importantly, the biological mechanism of the occurrence and treatment of esophageal stricture related to EEB damage and restore shall be clarified with more scientific evidence.

In recent years, more and more scholars have attempted to compensate for the re-epithelialization of mucosa with the injured EEB through tissue engineering technologies. For example, researchers injected the adipose mesenchymal stem cells (MSCs) into the post-ESD region of dogs, achieving45% of the mean degrees of mucosal constriction compared with 76% in the blank control (*p* < 0.008). The number of submucosal micro-vessels was also more than that in the blank control animals. Sure, a good blood supplement provides a suitable environment for epithelium repair [139]. Similar results were discovered in the porcine model using keratinocytes from the oral mucosa as the injected cells. The lesion in the keratinocyte implanted animal was covered by epithelial cells. The luminal surface was flat, with no ulceration taking place. Scarring and stricture were observed in the control group after two weeks post-EMR surgery [140].

The cell density and the exact location with cell injection are not able to be precisely control due to easily running off for the cell suspension. Some biological materials, such as amniotic membrane or decellularized matrix, etc., were studied as cell carriers. Cells were seeded on these materials to make cell patches. Nishida et *al*. reported that cell sheets could reconstruct the cornea, and the patients recovered their sight [141]. Ohki et al. fabricated the cell patches containing autologous oral mucosal cells cultured in temperature-responsive dishes. These patches were then transplanted onto mucosa wounds caused by ESD in the dog model. Complete healing of the mucosa was observed after the cell patches were placed for four weeks, whereas severe inflammation existed in blank control [142]. The same results were achieved in a porcine model, where endoscopic techniques placed the cell patches on the post-ESD area. Early re-epithelialization and moderate fibrosis in the muscle were observed in the transplanted animals, while all pigs in the blank control showed stricture occurrence [143]. Recently, a biosynthetic material with collagen vitrigel to make cellular patches (CVP) was studied by Aoki et al. They placed cellular sheets at the post-ESD site in pigs, inducing a re-epithelialization of the mucosa and reducing the hyperfibrosis, compared with a blank control group [144]. Research on animals has shown that mucosal re-epithelialization to improve EEB functionality aids in mitigating or forestalling pathological strictures. However, such animal trials, serving as anticipatory foundational studies, possess certain limitations, even as they endeavor to simulate actual clinical conditions. To further confirm these results, large-scale human clinical trials are necessary, considering the distinct differences in living conditions between animals and humans.

**Fig. 5:**
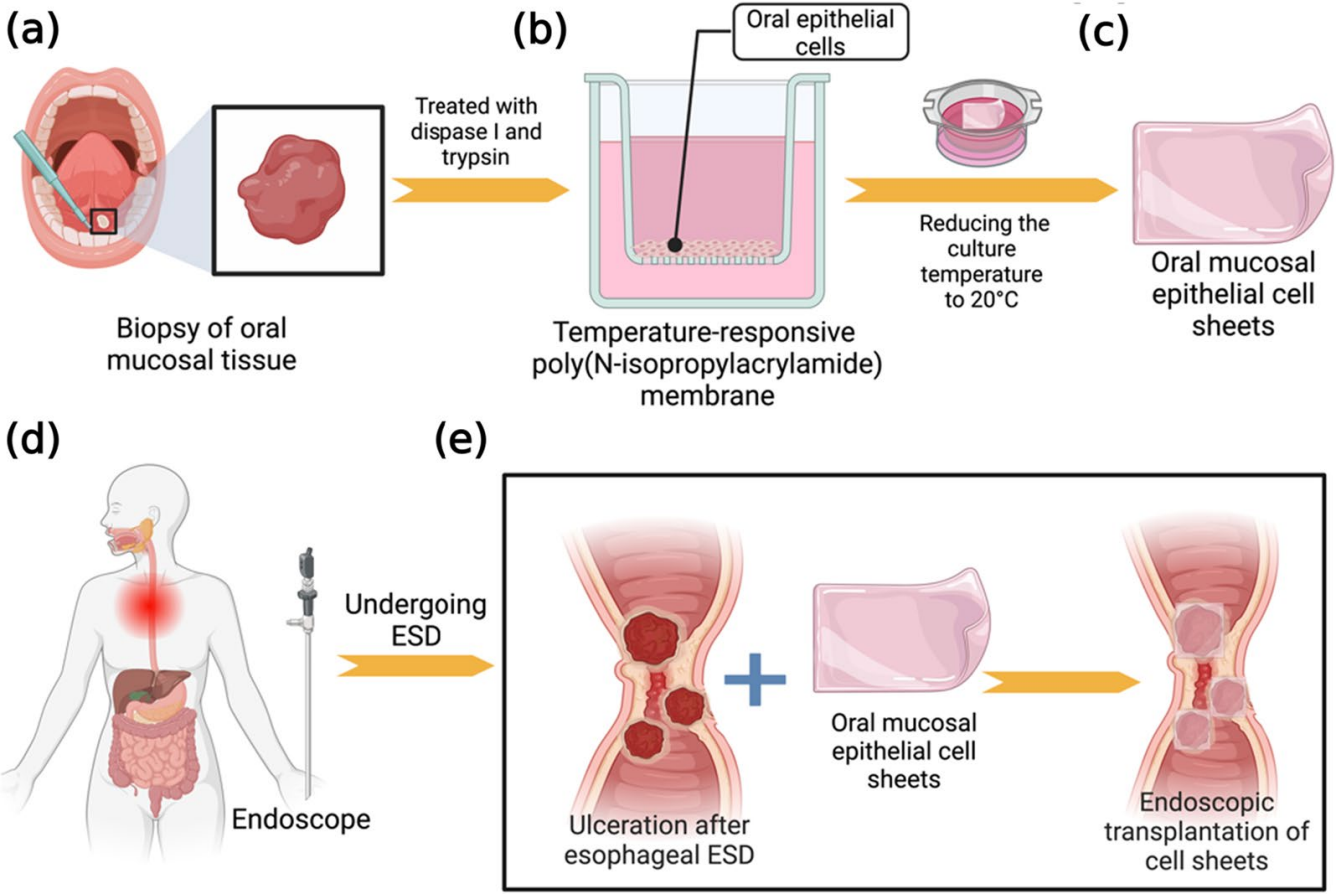
Treatment of the artificial ulceration after esophageal ESD by transplantation of autologous oral mucosal epithelial cell sheets fabricated on temperature-responsive culture inserts. **a** Biopsy specimens were taken from the patient’s own oral, buccal mucosal tissue. Oral epithelial cells were isolated from the tissue by dispase I and trypsin. **b** The epithelial cells were seeded onto temperature-responsive culture inserts without a 3T3 feeder layer and cultured with autologous serum for 16 days at 37 °C. **c** Oral mucosal epithelial cell sheets were harvested by reducing the culture temperature to 20 °C. **d**, **e** Autologous oral mucosal epithelial cell sheets on a support membrane were transplanted with endoscopic forceps onto the bed of the esophageal ulceration immediately after ESD (diagrammatically displayed by us according to the work of literature [119])

Correspondingly, clinical studies make less progress in this disease due to many ambiguities in the pathology and treatment mechanism. In a single-institute study, Ohki et al. investigated the potential of transplanting tissue-engineered cell sheets made from patients' own oral mucosal epithelial cells to avert post-ESD esophageal strictures. They gathered oral mucosal cells from 9 patients with non-deep esophageal cancers, cultivated them into cell sheets, and then transferred these sheets endoscopically to the surgical sites (Fig. 5). Monitoring through weekly endoscopies continued until the healing process was complete. The results showed a successful transplant and complete healing within an average timeframe of 3.5 weeks, with no subsequent complications like dysphagia or narrowing of the esophagus. This method, which involves no sutures and uses the patient's own cells, appears to safely and effectively promote healing after ESD, potentially preventing undesirable postoperative constriction and elevating patient quality of life. However, the study suggests that further investigation is needed to confirm its preventive capabilities against stricture formation [145]. Recently, researchers transplanted epithelial cell sheets to the post-operation area of patients who had congenital esophageal atresia. Six months later, the patient was aware of a reduction in dysphagia. And the intervals between endoscopic balloon dilatation were extended twice as much as the blank control group [146].

The efficacy of cellular patches in warding off esophageal strictures clarifies the connection between EEB deficits and the development of these strictures, providing a glimmer of hope for affected patients. However, the application of cellular patches as a treatment is still contested. Moreover, the small sample sizes in these clinical trials do not provide enough evidence to assert that cellular patches are suitable for widespread mucosal debridement in ESD or for patients with esophageal stricture.

### Cross-talking of multiple mechanisms

Esophageal stricture is often a parallel or cross-cascade result from multiple biologic mechanisms. The studies above suggest that fibrosis, inflammation, or EEB damage is not the independent individual etiology to induce esophageal stricture [147–152]. Kasagi et al*.* and Rochman*et al.* observed that some genes involved in barrier function are lost in EoE, triggering the inflammation progress. The abnormalities in gene or gene transcription/translation might delay EEB repair, contributing to wrong esophageal cell differentiation and even disease induction [149, 153]. Those with long-standing inflammation develop fibrosis in the esophagus at the mucosa and submucosa site. As a result, the esophagus becomes stiff, and hence the stricture develops [154, 155].

Histologically, it can be found that there is a mechanism for each link from the surface to the deeper layers, i.e., damage to the barrier—persistent inflammatory stress—excessive fibrosis. Harmful substances from digested food or the sick tissue weakened junction between mucosal epithelial cells, abnormally enlarged cell–cell gap, and disturbed translation of cornified envelope proteins. Consequently, the normal EEB is impaired to let those harmful substances attack deeper tissues.

**Fig. 6:**
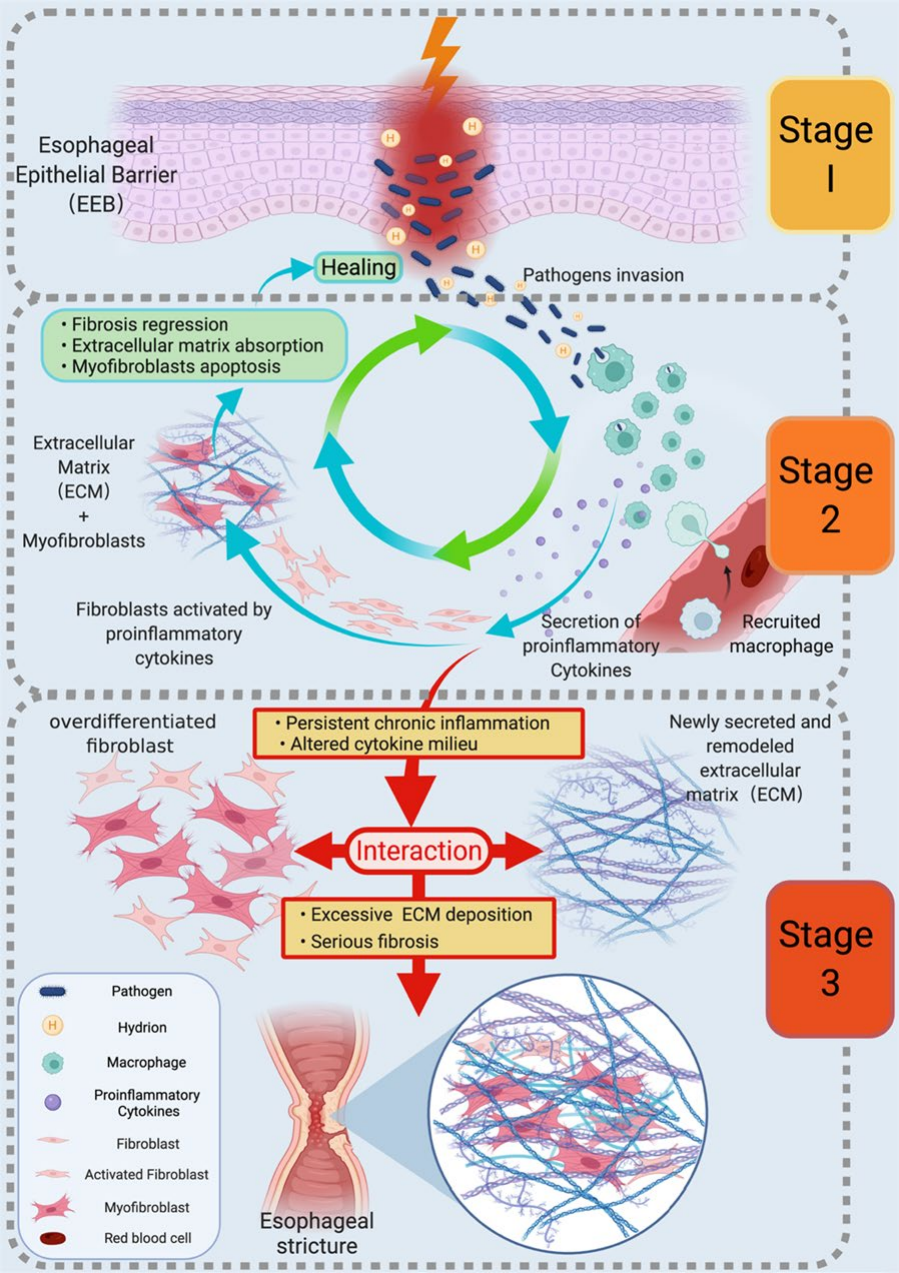
Schematic diagram of multiple crossed mechanisms by which the stricture in the esophagus takes place. The three stages of damage to the barrier—persistence of the inflammatory stress response—excessive fibrosis

Many diseases in the esophagus, such as EoE, GERD, and BE, start from the impaired EEB structure [130, 156–159]. As harmful substances attack the deeper tissue, the esophageal epithelium becomes severely damaged, thereby the body begins to initiate the inflammatory response. Macrophages start to work, and with the recruitment of macrophages, the aggregated macrophages begin to engulf harmful substances and release a large number of inflammatory factors and cytokines, which continuously stimulate fibroblasts to differentiate into myofibroblasts to repair damaged tissues. The duration of the inflammation is significantly essential, as the inflammatory response plays an active role in tissue repair. Once the inflammation progresses into chronic inflammation, the excessive differentiation of fibroblasts into myofibroblasts will lead to excessive secretion of cytokines and finally lead to the occurrence of severe fibrosis. As a result, the esophagus becomes stiff, and the stricture is eventually induced. These crossed multi-mechanisms by which the esophageal stricture takes place were schematically drawn in Fig. 6. We believe that any medicines or biomaterials which can interfere with these mechanisms will be the therapies for esophageal stricture.

In recent years, more and more scholars in the area of tissue engineering are trying to find a kind of biocompatible biomaterial that can not only inhibit inflammation and fibrosis but also facilitate epithelium regeneration. Some researchers have found that decellularized human amniotic membranes had all three functions: anti-inflammation, anti-fibrosis, and promotion of epithelialization. For example, Chen et al*.* found that decellularized human amniotic membranes could achieve an anti-fibrosis effect by inhibiting MMP-2 [160]. Oba et al. attached decellularized human amniotic membranes to the wound area of burn patients and found that it had anti-inflammatory and antibacterial effects, measured by immunohistochemical staining and corresponding protein detection [161]. These studies give supports of our hypothesis that esophageal strictures are caused by a disease in which multiple mechanisms exist in parallel and interact with each other.

## Conclusion

The burden of esophageal stricture, whether from disease or treatment like ESD/EMR, is becoming more manageable as we unravel its complex mechanisms. We see it as a dynamic process involving multiple stages: initial EEB damage, inflammatory escalation, and final fibrotic closure.

Therapeutically, we have pinpointed interventions including steroids, MMC, and cell sheet technology for the distinct pathogenic mechanisms of esophageal stricture. However, these studies stand to gain from the expansion into larger multicenter clinical trials to thoroughly ascertain their therapeutic value in practice. Our findings suggest that the condition of esophageal narrowing is due to intertwined mechanisms, and new treatment avenues like tissue engineering could offer a more comprehensive approach. The full promise of blending such regenerative approaches with gene editing remains an exciting prospect for future observation.

Nevertheless, the full effectiveness of these therapies and their applicability to extensive tissue damage have yet to be determined. As our understanding grows, especially in the fields of genetics and proteomics, we anticipate the identification of key genes and regulatory elements crucial for epithelial protection and recovery. This knowledge is expected to lead to new treatments that better alleviate patient suffering.

## Data Availability

All data generated or analysed during this study are included in this published article [and its additional information files].
